# Supplementary material for: Fluorescence Lifetime Imaging for Quantification of Targeted Drug Delivery in Varying Tumor Microenvironments
Source: Adv Sci (Weinh). 2024 Nov 27;12(3):2403253. doi: 10.1002/advs.202403253 (PMC11744649; doi:10.1002/advs.202403253)
Supplement: Supplementary file 1 — Supporting Information [file ADVS-12-2403253-s001.docx]

**SUPPLEMENTARY FIGURES**

**
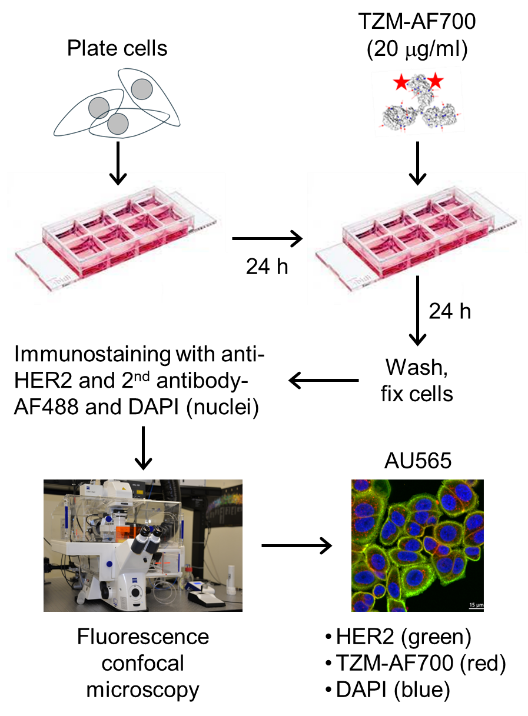
Figure S1.** Schematic representation of cell-based uptake and immunofluorescence assay.

**
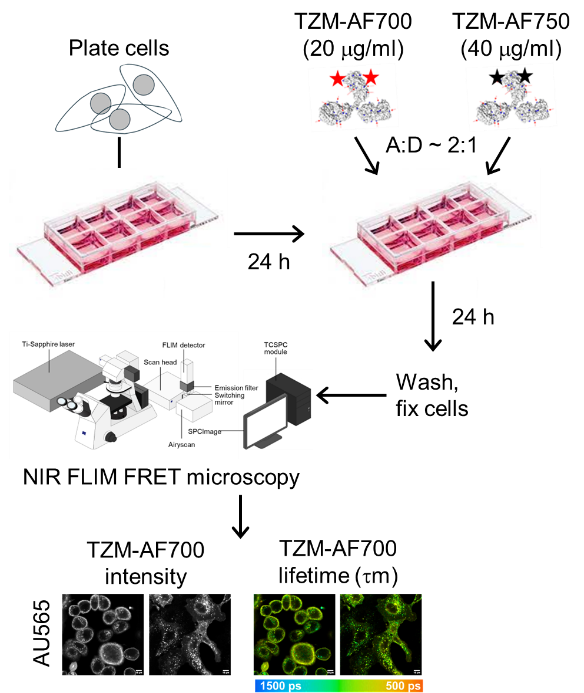
Figure S2.** Schematic representation of cell-based uptake of NIR-labeled TZM FRET pair and fluorescence lifetime microscopy (FLIM) assay.

**
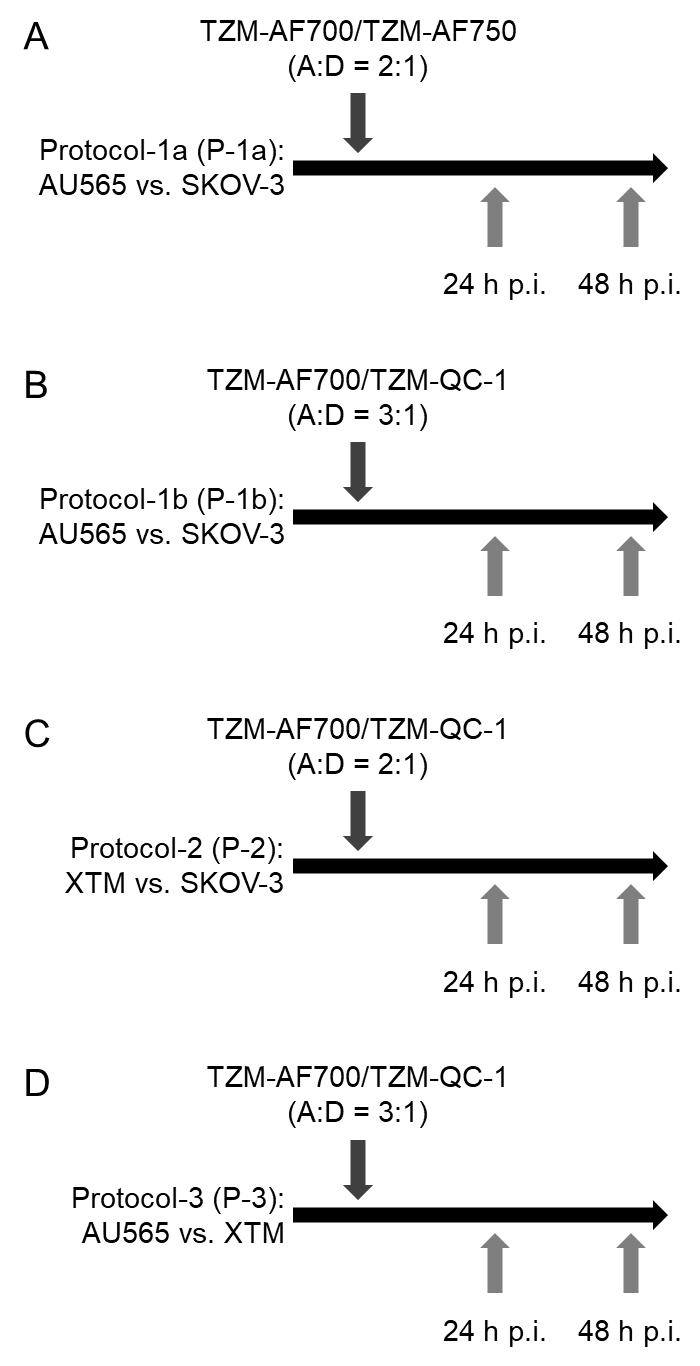
Figure S3.** Injection and *in vivo* imaging protocols for AU565, XTM, and SKOV-3 tumor xenografts-bearing mice: **(A)** **Protocol 1a (P-1a)**, mice bearing AU565 and SKOV-3 tumor xenografts were injected intravenously with TZM-AF700/TZM-AF750 (A:D 2:1) and live intact mice MFLI data was captured at 24 h and 48 h p.i.; **(B)** **Protocol-1b (P-1b),** mice bearing AU565 and SKOV-3 tumor xenografts were injected intravenously with TZM-AF700/TZM-QC-1 (A:D 3:1) and live intact animal MFLI data was captured at 24 h and 48 h p.i.; **(C)** **Protocol 2 (P-2)**, mice bearing XTM and SKOV-3 tumor xenografts were injected intravenously with TZM-AF700/TZM-QC-1 (A:D 2:1) and live intact animal MFLI data was captured at 24 h and 48 h p.i.; **(D)** **Protocol-3 (P-3)**, mice bearing AU565 and XTM tumor xenografts were injected intravenously with TZM-AF700/TZM-QC-1 (A:D 3:1) and live intact animal MFLI data was captured at 24 h and 48 h p.i..

**
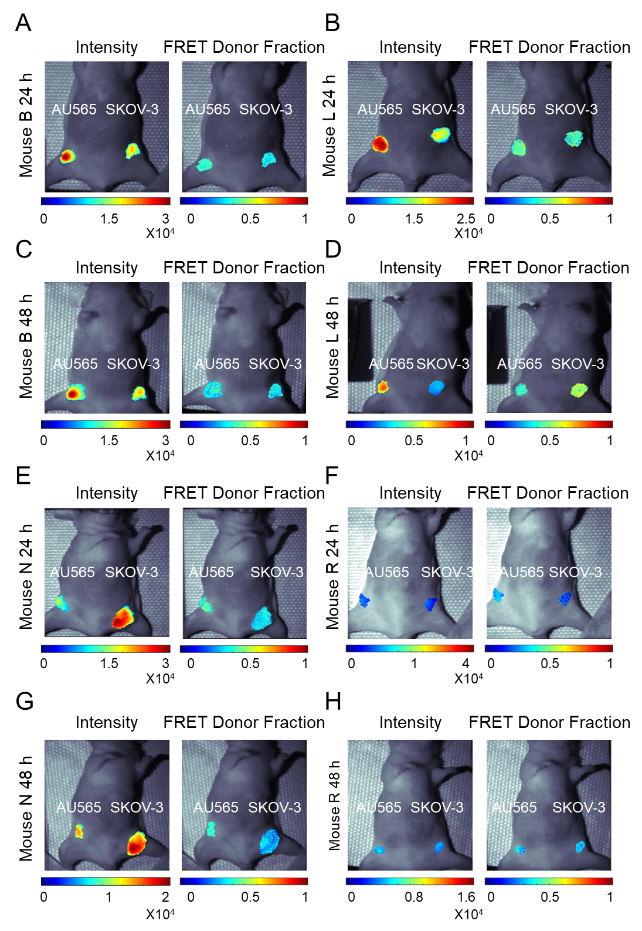
Figure S4.** MFLI FRET *in vivo* imaging in nude mice carrying AU565 and SKOV-3 tumor xenografts (Protocol P-1a). **(A-H)** Photomicrographs show TZM donor maximum intensity ROIs (both soluble and bound probe) and FD% map (bound and internalized probe) in TZM–AF700/ TZM–AF750 treated AU565 and SKOV-3 tumors (T), at 24 h p.i. (**A-B, E-F**) and 48 h p.i. (**C-D and G-H**); TZM–AF700 and TZM–AF750 treated AU565, n= 5; SKOV-3, n= 5, n= number of tumors analyzed per group.

**
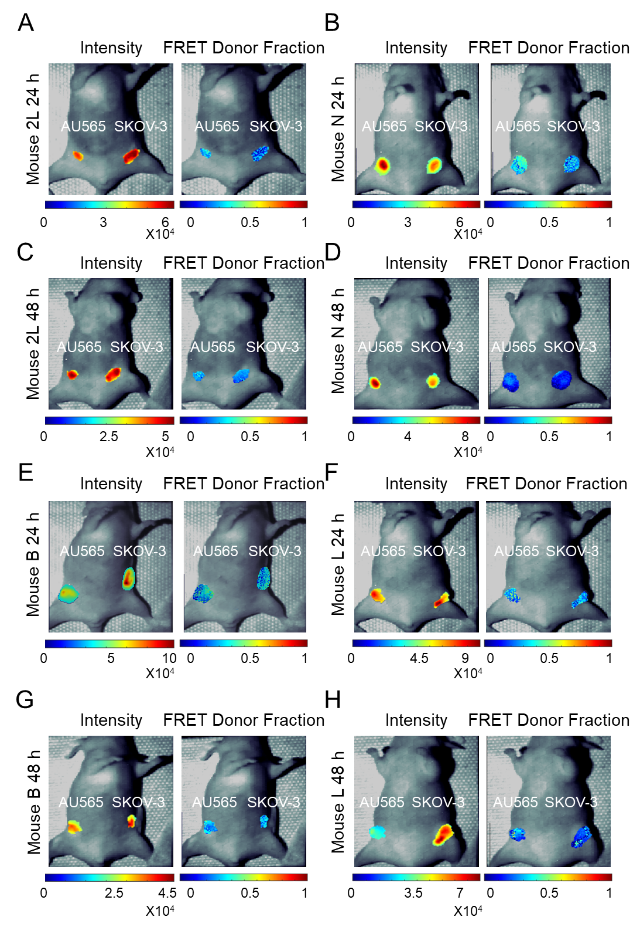
Figure S5.** MFLI FRET *in vivo* imaging in nude mice carrying AU565 and SKOV-3 tumor xenografts (Protocol P-1b). **(A-H)** Photomicrographs show TZM donor maximum intensity ROIs (both soluble and bound probe) and FD% map (bound and internalized probe) in TZM–AF700/ TZM–QC-1 treated AU565 and SKOV-3 tumors (T), at 24 h p.i. (**A-B and E-F**) and 48 h p.i. (**C-D and G-H**); TZM–AF700 and TZM–QC-1 treated AU565, n= 4; SKOV-3, n= 4, n= number of tumors analyzed per group.

**
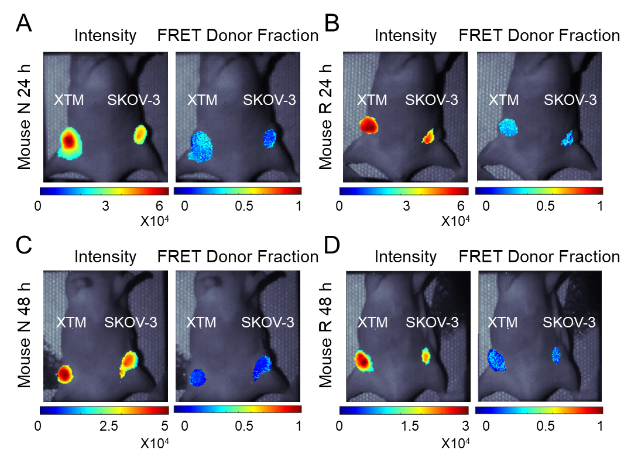
**

**Figure S6.** MFLI FRET *in vivo* imaging in nude mice carrying XTM and SKOV-3 tumor xenografts (Protocol P-2). **(A-D)** Photomicrographs show TZM donor maximum intensity (both soluble and bound probe) and FD% map (bound and internalized probe) for tumor ROIs in TZM–AF700/ TZM–QC-1 treated XTM and SKOV-3 tumors (T), at 24 h p.i. **(A-B)** and 48 h **(C-D)** p.i. TZM–AF700 and TZM–QC-1 treated XTM, n= 3; SKOV-3, n=3. n= number of tumors analyzed per group.

**
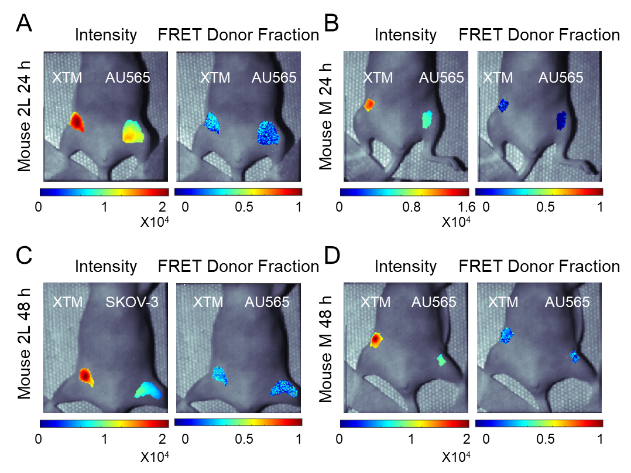
Figure S7.** MFLI FRET *in vivo* imaging in nude mice carrying AU565 and XTM tumor xenografts (Protocol P-3). **(A-B)** Photomicrographs show TZM donor maximum intensity (both soluble and bound probe) and FD% map (bound and internalized probe) for tumor ROIs in TZM–AF700/ TZM–QC-1 treated AU565 and XTM tumors (T), at 24 h p.i. (**A-B**) and 48 h p.i. (**C-D**); TZM–AF700 and TZM–QC-1 treated XTM, n= 3; AU565, n=3. n= number of tumors analyzed per group.

**Supplementary Tables**

**Supplementary Table S1.** Experimental reagents used.

| Supplementary Table 1. List of reagents, supplies and instruments | | |
| --- | --- | --- |
| Product | Company | Catalogue No |
| Amicon Ultra-4-centrifugal filter units (MWCO 30 kDA) | Sigma Aldrich, St. Louis, MO | Z648035 |
| Anti-HER2 primary antibody | Thermo Fisher Scientific Inc, Waltham, MA | MA5-12759 |
| Trastuzumab (Anti-Human HER2, Humanized Antibody) | MedChemExpress, NJ | HY-P9907 |
| HER2/ErbB2 (29D8) Rabbit mAb | Cell Signaling, Technology | 2165 |
| Monoclonal rabbit CD31 | Cell Signaling Technology, Inc., Danvers, MA | 77699 |
| Rabbit monoclonal anti-TZM | R&D Systems, Bio-Techne, Minneapolis, MN | MAB95471-100 |
| Goat anti-mouse labeled with Alexa Fluor 488 | Abcam Inc | ab181289 |
| Alexa Fluor 700 NHS ester | Thermo Fisher Scientific Inc, Waltham, MA | A20110 |
| Alexa Fluor 750 NHS ester | Thermo Fisher Scientific Inc, Waltham, MA | A37575 |
| IRDye® QC-1 NHS Ester | Lincoln, NB, USA | 929-70030 |
| Olympus BX40 microscope equipped with an Infinity 3 camera | Lumenera Inc., Ottawa, ON, Canada | BX40-B |
| BME001-05 | R&D Systems Inc, Minneapolis, MN, USA | BME001-01 |
| Nude mice, CrTac: NCr-Foxn1nu | Taconic Biosciences, Rensselaer, NY | NCRNU-F |
| Vectastain ABC Elite kit | Vector Labs, Burlingame, CA | PK-6100 |
| Vectro NovaRED | Vector Labs, Burlingame, CA | SK-4800 |
| Methyl Green | Sigma Aldrich, St. Louis, MO | M8884 |
| AU565 | ATCC, Manassa, VA | CRL-2351 |
| SKOV-3 | ATCC, Manassa, VA | HTB-77 |
| RPMI 1640 | Thermo Fisher Scientific Inc, Waltham, MA | 11875093 |
| McCoy’s media | Thermo Fisher Scientific Inc, Waltham, MA | 16600082 |
| Hanks' Balanced Salt Solution (HBSS) | Thermo Fisher Scientific Inc, Waltham, MA | 88284 |
| µ-Slide 8 Well | Ibidi, Fitchburg, WI, | 80826 |
| Fetal bovine serum | ATCC, Manassa, VA | 30-2021 |
| HEPES | Thermo Fisher Scientific Inc, Waltham, MA, | 15630080 |
| Penicillin/Streptomycin | Thermo Fisher Scientific Inc, Waltham, MA, | 15070063 |
| Beckman DU-640 Spectrophotometer | GMI | SKU: 8043-30-1090 |
| Digital micromirror (DMD) device | Texas Instruments, TX | DLi 4110 |
| Titanium/ Sapphire laser, Chameleon Ultra II | Coherent, Inc., Santa Clara, CA | NA |
| Mai Tai Ultrafast Laser | Spectra-Physics, CA | NA |
| SPCImage NG Data Analysis Software | Becker & Hickl GmbH, Berlin, Germany | NA |
| Intensified CCD (ICCD) camera | Picostar HR, Lasision GmbH, Bielefeld, Germany | NA |

**Supplementary Table S2.** Statistical analysis for data presented in **Figure 2D-G**

| Supplementary Table 2. Statistical analysis for data presented in Figure 2 D-G | | | | |
| --- | --- | --- | --- | --- |
| Acceptor to Donor ratio (A:D) | Figure 2 D | Figure 2 E | Figure 2 F | Figure 2 G |
| 0:1 | p =   1.38e-9 | p =   1.01e-6 | p =   0.5 | p =   0.495262 |
| 1:1 | p =   0.003519 | p =   7.0e-5 | p =   0.000129 | p =   0.343776 |
| 2:1 | p =   9.47e-6 | p =   1.12e-7 | p =   0.100669 | p =   0.028397 |
| 3:1 | p =   0.135675 | p =   0.016337 | p =   7.3e-6 | p =   1.44e-6 |
| Two sample T-TEST; p-value ≤ 0.05 is significant. | | | | |

**Supplementary Table S3 and S4.** Statistical analysis for data presented in **Figure 4C-D**

| Supplementary Table 3. Statistical analysis for data presented in Figure 4 C-D | | | | | | | |
| --- | --- | --- | --- | --- | --- | --- | --- |
| TZM-AF700/TZM-AF750 (24 h) | | | | TZM-AF700/TZM-AF750 (48 h) | | | |
| Cells | N total (pixels) | Mean | Standard Deviation |  | N total (pixels) | Mean | Standard Deviation |
| AU565 | 360 | 0.4276 | 0.040045 | AU565 | 1316 | 0.4015 | 0.0779 |
| SKOV-3 | 1638 | 0.357375 | 0.047042 | SKOV-3 | 1826 | 0.3331 | 0.0727 |
| Two sample T-TEST; p-value 5.7E-261; p-value ≤ 0.05 is significant. | | | | Two sample T-TEST; p-value 6E-261; p-value ≤ 0.05 is significant. | | | |

| Supplementary Table 4. Statistical analysis for data presented in Figure 4 C-D | | | | | | | |
| --- | --- | --- | --- | --- | --- | --- | --- |
| TZM-AF700/TZM-QC-1 (24 h) | | | | TZM-AF700/TZM-QC-1 (48 h) | | | |
| Cells | N total (pixels) | Mean | Standard Deviation |  | N total (pixels) | Mean | Standard Deviation |
| AU565 | 360 | 0.3227 | 0.0743 | AU565 | 322 | 0.196 | 0.097 |
| SKOV-3 | 305 | 0.2871 | 0.062 | SKOV-3 | 384 | 0.1797 | 0.0906 |
| Two sample T-TEST; p-value 3.52E-11; p-value ≤ 0.05 is significant. | | | | Two sample T-TEST; p-value 0.0223; p-value ≤ 0.05 is significant. | | | |

**Supplementary Table S5.** Statistical analysis for data presented in **Figure 6B**

| Supplementary Table 5. Statistical analysis for data presented in Figure 6 B | | | | | | | |
| --- | --- | --- | --- | --- | --- | --- | --- |
| TZM-AF700/TZM-QC-1 (24 h) | | | | TZM-AF700/TZM-QC-1 (48 h) | | | |
|  | N total (pixels) | Mean | Standard Deviation |  | N total (pixels) | Mean | Standard Deviation |
| XTM | 611 | 0.2993 | 0.0577 | XTM | 349 | 0.257 | 0.0578 |
| SKOV-3 | 642 | 0.2421 | 0.0806 | SKOV-3 | 442 | 0.2286 | 0.0743 |
| Two sample T-TEST; p-value 6.87827E-44; p-value ≤ 0.05 is significant. | | | | Two sample T-TEST; p-value 2.229E-09; p-value ≤ 0.05 is significant. | | | |

**Supplementary Table S6.** Statistical analysis for data presented in **Figure 7B**

| Supplementary Table 6. Statistical analysis for data presented in Figure 7 B | | | | | | | |
| --- | --- | --- | --- | --- | --- | --- | --- |
| TZM-AF700/TZM-QC-1 (24 h) | | | | TZM-AF700/TZM-QC-1 (48 h) | | | |
|  | N total (pixels) | Mean | Standard Deviation |  | N total (pixels) | Mean | Standard Deviation |
| AU565 | 483 | 2.33E-01 | 0.07919 | AU565 | 151 | 0.2202 | 0.0966 |
| XTM | 487 | 2.43E-01 | 0.0876 | XTM | 151 | 0.2362 | 0.0626 |
| Two sample T-TEST; p-value 0.0707; p-value ≤ 0.05 is significant. | | | | Two sample T-TEST; p-value 0.0871; p-value ≤ 0.05 is significant. | | | |
